# Use of nutritional information: analysing clusters of consumers who intend to eat healthily

**DOI:** 10.1017/jns.2019.13

**Published:** 2019-04-29

**Authors:** Vincent J. van Buul, Catherine A. W. Bolman, Fred J. P. H. Brouns, Lilian Lechner

**Affiliations:** 1 Faculty of Psychology and Educational Sciences, Open University of the Netherlands, PO Box 2960, 6401 DL Heerlen, the Netherlands; 2 Faculty of Health, Medicine and Life Sciences, Department of Human Biology, NUTRIM-School of Translational Research in Nutrition and Metabolism, Maastricht University, 6200 MD Maastricht, the Netherlands

**Keywords:** Food choice, MouselabWEB, Process tracing, Health action process approach, Nutrition information, HAPA, health action process approach, NLS, Nutritional Literacy Scale

## Abstract

Consumers intending to eat healthily should consult available information on the energy, salt, sugar and saturated fat content of foods. Some consumers, however, do this more than others do. The objective of this research was to identify distinct subgroups within the group of consumers who intend to eat healthily, segmented according to the timing and frequency of their use of information about energy, salt, sugar and saturated fat. Furthermore, we analysed whether consulting this information actually led to healthier food choices. Data on use of specific nutritional information in a computerised task in which participants made multiple dichotomous food choices (e.g. high-fat *v.* low-fat cheese) were recorded from 240 participants using process tracing software. Participants could view nutritional information by hovering the mouse over specific areas of the screen. We found three clusters of participants based on use of information about energy, salt, sugar and saturated fat: low, medium and high information users. There was a between-clusters difference in how often the healthy option was chosen (88·95 % with high information *v.* 67·17 % with low information usage). Presence in the medium and high information clusters was partially predicted by perceived self-efficacy in making healthy choices. It appears that some consumers are very confident of their ability to make healthy choices, which is a reason for making less use of nutritional information prior to making food choices and may result in unhealthy choices. Our findings improve understanding of the conditions needed to develop effective interventions targeted at health-conscious consumers.

In the last decades, it has become overwhelmingly clear that regular intake of foods high in energy and with added salt, sugar and saturated fat are among the key risk factors for developing overweight, metabolic disorders and related diseases^(^^1^^)^. Often, food high in one or more of these four attributes is an industrial, highly processed product and such food can be considered troublesome from social, cultural, economic, political and environmental points of view, as outlined in a recent commentary^(^^2^^)^. Because of this, consumers should be encouraged to consult on-pack information about the nutrient and energy content of foods^(^^3^^)^. Consumers are being urged to choose foods with a better nutritional composition through front-of-pack nutrition claims (e.g. ‘low sugar’), public health campaigns (e.g. increasing consumer awareness of salt intake) and food reformulations affecting back-of-pack nutritional information (e.g. lowering saturated fat levels and/or energy of processed foods)^(^^4^^,^^5^^)^.

There has been an abundance of studies investigating the effects of incorporating nutritional information into food packaging on the food choices of various populations^(^^6^^–^^8^^)^ and there is a growing body of evidence that not all ways of presenting nutritional information to the consumer have similarly desirable effects on food choices. For instance, simplified, front-of-pack, single-nutrient claims, particularly ‘low-fat’, could be hindering the general public from making healthier choices, as research has shown that it could lead to underestimation of total energy^(^^9^^)^. In most of these studies towards the relationship between nutrition information and behaviour, the available nutrition information itself is manipulated between the groups that are studied, rather than measured and compared within a particular group^(^^10^^)^.

Multiple studies have already shown that there is considerable heterogeneity in how certain predictors (e.g. taste perceptions, ingredient awareness, presentation of nutritional information) affect how health-conscious consumers assess the healthiness of foods^(^^11^^–^^14^^)^. There have been few studies, however, focusing on how nutritional information, regardless of how it is presented, is used by consumers who intend to eat healthily and how it affects the healthiness of food choices. Individuals who have a strong intention to eat healthy foods are of great interest to public health researchers because they are motivated to eat healthily, which is a hard-to-convey prerequisite for true healthy eating behaviour, according to current health psychological theory^(^^15^^)^. It is only in the later stages of the process – as modelled by current theories – of translating intentions into behaviour that there appears to be a problem, with the intended healthy behaviour failing to emerge^(^^16^^)^.

Our main research objective, therefore, was to understand better the heterogeneity of this group, what their characteristics are, and whether they can be grouped in certain subgroups showing similar patterns concerning the consideration of nutrition information. We aimed to use this information to uncover demographic and psychosocial predictors of membership of the use-of-nutritional-information subgroups. These insights will help to optimally develop innovative, evidence-based, policies and interventions that will enhance people's ability to make healthy food choices^(^^17^^)^.

Building on our broad theoretical psychological model of the determinants of inadvertent unhealthy substitutive food choices from our earlier study^(^^18^^)^, the aim of the present study was to provide concrete suggestions about how nutritional behaviour can be improved through differentiating between consumers who intend to eat healthily on the basis of how they use nutritional information usage and psychosocial characteristics. This required a robust, careful assessment of use of nutritional information, as it has been suggested that simply distinguishing between ‘users’ and ‘non-users' of nutritional information is too simplistic to yield any meaningful conclusions^(^^19^^)^.

We based our examination of psychosocial variables that could differ between consumers on the health action process approach (HAPA) model, a social–cognitive model that describes the key stages and cognitions involved in acting on an intention^(^^20^^)^. In earlier studies^(^^18^^,^^21^^,^^22^^)^ this model provided an interesting framework of psychosocial variables that partially explained differences in food choice behaviour. The HAPA model combines insights from several theories to explain how people can become aware of their personal health and develop an intention to change it, and the dynamic process that follows the development of such an intention. According to the HAPA model, people can develop the intention to become healthier (motivation) and subsequently act on these intentions (volition). In the volition phase, one can distinguish between two groups of individuals: those who have not yet translated their intentions into action, and those that have. The model is useful for investigating the problem at hand because it splits the process of behavioural change into several stages.

In our research, we assume that consumers who have the intention to eat healthily recognise the risk of unhealthy food consumption. As such, they should understand the expected outcomes of changing behaviour, and are capable of exercising control of their actions^(^^23^^)^. Moreover, we assume that there are factors other than motivation and intention at play in the complex behaviours relating to food choice. Earlier studies have shown that nutritional literacy^(^^24^^,^^25^^)^, potentially exposure to food retailers^(^^26^^)^ and taste preferences^(^^9^^,^^27^^)^ have a profound influence on food choice behaviour and hence health. We chose to focus on nutritional literacy as it has emerged as a key component in the promotion and maintenance of healthy dietary practices. Although nutritional literacy is sometimes considered part of food literacy^(^^28^^,^^29^^)^, we focus on nutritional literacy defined as the capacity to obtain, process, and understand nutritional information and the skills needed to make appropriate nutritional decisions^(^^30^^)^. Food literacy is often defined more broadly and embraces components including food safety and food waste practices.

Given these considerations and the objective of our study, we posed the following research question:

Are there distinct subgroups within the group of consumers who intend to eat healthily, segmented by the time and frequency of their use of information about the energy, salt, sugar and saturated fat content of foods and, if so, what are the demographic, psychosocial, nutritional literacy and taste preference variables that differentiate between them?

Given that there has been evidence that using nutritional information has a positive effect on healthy choices in the general population, we hypothesised that a cluster of consumers who intend to eat healthily, who can be segmented on high usage of nutrition information, will make significantly more healthy food choices (hypothesis 1).

On the basis of the HAPA model, we hypothesised that there are differences in the levels of intention, action planning and/or self-efficacy between clusters of consumers who intend to eat healthily, related to their use of nutritional information. We predicted that the segment of consumers who made more use of nutritional information would also display a greater intention to eat healthily, engage in more planning and consider themselves to be more capable of making appropriate food choices (i.e. report greater self-efficacy) than segments with lower use of nutritional information (hypothesis 2).

To increase the explanatory power of our model we broadened its scope and included two additional predictors that were, on theoretical grounds, of potential relevance. We therefore posited that there would be differences between the nutritional literacy scores and/or taste preferences of clusters of consumers who intend to eat healthily, related to their use of nutritional information (hypothesis 3).

## Method

### Study design and participants

We conducted an online process tracing study and survey as part of an earlier study^(^^18^^)^. In this study, Dutch-speaking consumers who intended to eat healthily were asked to fill in an online questionnaire on their psychosocial and demographic characteristics, intention to eat healthily and their food choice behaviour in July 2016. Participants were recruited through an online panel (http://academicresearchpanel.com/). A random subset of members of this panel was invited to participate in the study by email. The introductory email included a link to a page that outlined the broad purpose of the study and information about inclusion criteria (i.e. >17 years of age, intending to eat healthily in the coming period, and use of a computer with a mouse (not a touchscreen device)). Informed consent was acquired by prompting participants to tick boxes behind four statements, to indicate that they understood the purpose of the study, had been informed of the inclusion criteria, understood that the data collected would not be linked to personal information and that they were free to drop out at any time without giving a reason. A total of 240 participants complied with the inclusion criteria, provided informed consent and completed all the questions and tasks in the online research. The mean age of participants was 51·65 (sd 13·72) years and their mean BMI was 25·42 (sd 4·03) kg/m^2^; 148 (61·7 %) were female.

### Measurement of variables

As detailed in our earlier work^(^^18^^)^, we developed an online questionnaire in Dutch that included variables designed to measure all the HAPA model constructs: intention, self-efficacy, action planning and coping planning. Responses to items were given using a seven-point scale ranging from completely disagree (1) to completely agree (7). The intention to eat healthily construct was measured by translating and adapting items from Schwarzer & Renner^(^^31^^)^, e.g. ‘I intend to eat healthful foods over the next months’. The self-efficacy construct was also measured with items adapted from Schwarzer & Renner^(^^31^^)^, e.g. ‘I can manage to stick to healthful food even if I have limited time’. Action planning and coping planning were measured by translating and combining items used in a variety of HAPA related studies^(^^32^^–^^34^^)^, e.g. ‘I have a detailed plan how to respond when someone offers me an unhealthy snack’. Furthermore, we asked participants about their demographic status and included items on psychosocial factors.

Nutritional literacy was one of the important constructs in this study^(^^35^^,^^36^^)^. As introduced, nutritional literacy is defined as the capacity to obtain, process, and understand nutritional information and the skills needed to make appropriate nutritional decisions Several nutritional literacy scales have been developed and reviewed recently^(^^24^^,^^29^^,^^37^^)^ and the Nutritional Literacy Scale (NLS) has been praised by dietitians and confirmed suitable in multiple studies^(^^30^^,^^35^^,^^36^^,^^38^^)^. The NLS consists of a sequence of statements in which a part of a sentence is left blank (e.g. ‘Calcium is ____ for bone health’. Participants are presented with four options (e.g. ‘essential’, ‘osteoporosis’, ‘expensive’, ‘prescription’) and asked to pick the one that best completes the statement. We used the NLS as guidance in the development of our questionnaire in which we translated and adapted (e.g. changing ounces to grams) the original items to fit the Dutch situation. Six of the original twenty-eight items were not included because of a poor fit to the nutritional literacy construct as defined in this study (e.g. questions about the price of healthy foods and weed-control techniques used in production of organic foods).

### Measurement of food choices and use of nutritional information

**Fig. 1.**
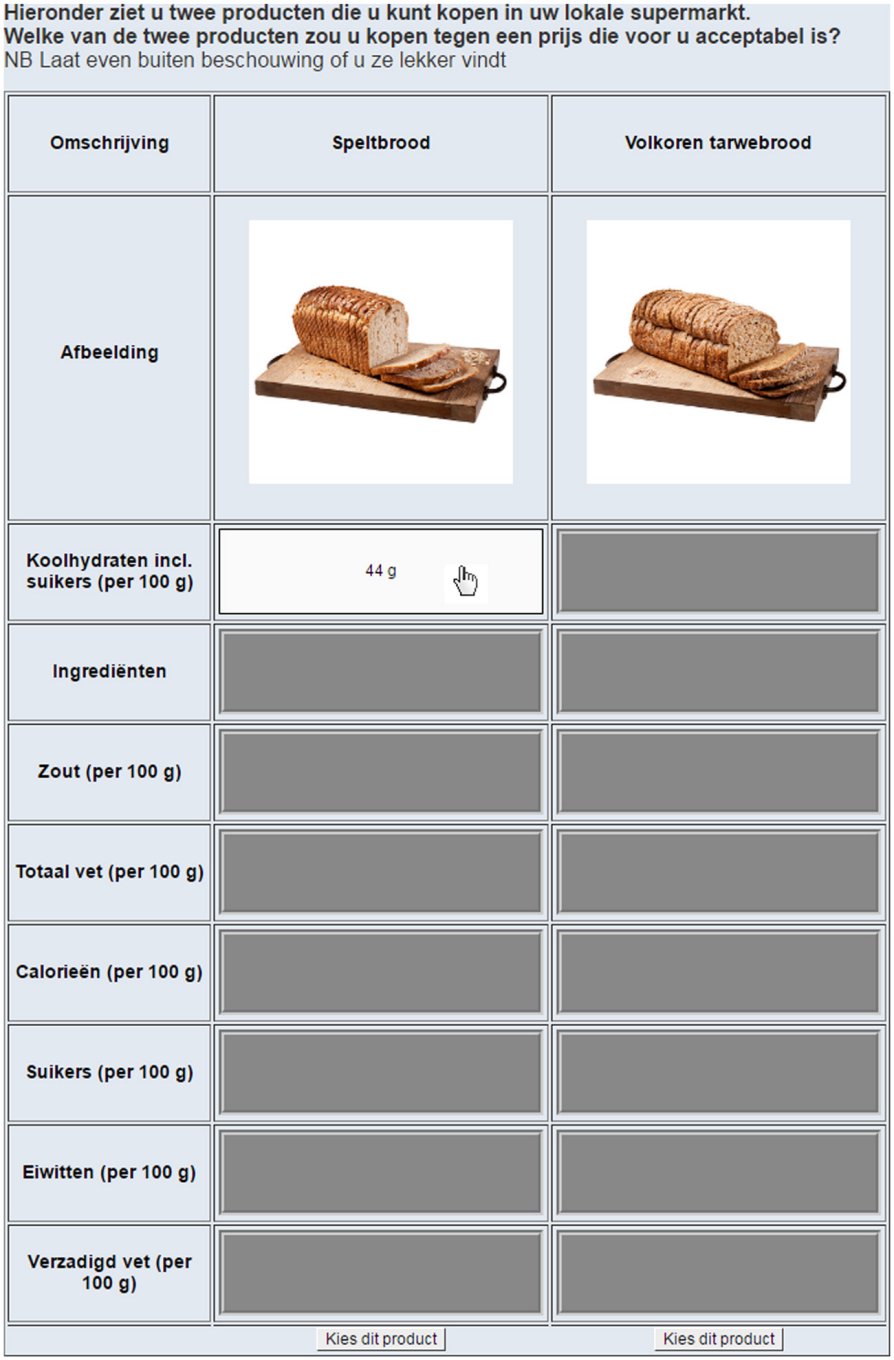
Screenshot of a choice matrix provided in MouselabWEB, as also used in our earlier research^(^^1^,^8^^)^.

Drawing on a 2007 computer-based experiment on food choices^(^^39^^)^, we used an open-source programme to quantify food choice behaviour (MouselabWEB 1.00 beta^(^^40^^)^). Like another computer-based experiment using pictures of canteen lunches^(^^41^^)^, we investigated what nutritional information was used to make choices. As in our earlier work^(^^18^^)^, we prompted participants to choose between nine different pairs of common food products that are found in supermarkets. Participants were shown the name and picture of each product and had the option of viewing eight additional attributes in a matrix format, similar to that used for food labels. Participants were asked which of the two products they would purchase assuming that the price of both was acceptable to them and were instructed not to base their decisions on taste (see Fig. 1).

At the beginning of each choice, all pieces of nutrition information on product attributes were not visible to the participant (i.e. energy, fat content, protein content, etc.). The picture and the name of the product were visible. Participants could make information visible by opening a cell by moving the cursor over it; the cell closed when the cursor was moved away. The MouselabWEB software tracked both the frequency with which these cells were opened and the time for which they remained open and stored it on the university server on which the online questionnaire was hosted.

The matrix consisted of two columns, each representing one food product. Each column displayed the name of the product, an image of it, and eight nutritional attributes (ingredient declaration, energy content, total carbohydrate content, sugar content, total fat content, saturated fat content, protein content and salt content). The position of the foods in the columns (left/right) was randomised across choice trials and participants. The row in which the closed attributes appeared was also counterbalanced, but remained linked to the appropriate column. Participants read detailed instructions about how to use MouselabWEB and conducted a guided practice trial before starting the food choice task^(^^41^^)^.

We included the data in our analysis on nutrition information considered only when participants opened a cell for more than 100 ms. We expected acquisitions <100 ms not to be read and comprehended by the participants, based on eye-fixation literature^(^^42^^)^. Such data points were the result of involuntary or accidental openings when scrolling over the page. Additionally, like our earlier research^(^^18^^)^, we excluded the choices that participants made for products that they were allergic to, or had a medical condition prohibiting consumption. These choices were not counted towards their score of healthy choices made.

The nine food choices were picked from the website of the Dutch Centre for Nutrition^(^^43^^)^ (see Table 1). This institute uses the so-called Wheel of Five (Dutch: De Schijf van Vijf), a food products–healthy choices information graphic, to give straightforward nutrition advice. The institute's website provides food choice recommendations. The nine choices that we used were all listed on the website as examples of what foods can be easily substituted with a similar product. In Table 1, we included the rationale behind why the two choices differ in terms of healthiness.

**Table 1.** The eighteen comparable food products (nine dichotomous choices) presented to participants, as presented earlier in Van Buul *et al.*^(^^18^^)^

Choice	Unhealthy choice*	Healthy choice*	Rationale
1	Gouda cheese 48+†	Gouda cheese 30+†	Cheeses that have the ‘48+’ indication have a higher saturated fat content compared with cheeses with the ‘30+’ indication
2	Coconut oil	Olive oil	Olive oil has less saturated fat than coconut oil
3	Spelt bread (refined)	Whole-wheat bread	Bread made from refined flour (regardless of species of wheat used) contains less fibre
4	Canned string beans (with added salt)	Frozen string beans	Salt and sometimes also sugar are often added to vegetables in cans, not to frozen vegetables
5	Culinary pork loin (injected with water and additives)	Pork loin	There is a link between eating processed meat and a higher risk of stroke, type 2 diabetes and colon cancer
6	Calvé peanut butter	100 % Peanut butter	Calvé peanut butter contains more salt, sugar and saturated fats
7	Santa Maria Extra Fine Selection Thai Red Curry	Original Spices by Jonnie Boer Thai Red Curry	The Santa Maria spice mix has added salt, while the Original Spices by Jonnie Boer does not
8	Salted full-cream butter	Liquid baking fat (from vegetable oils)	In comparison, there are more saturated fats in hard fats
9	Feta 45+	Goat cheese 45+	The feta cheese contains more salt than the alternatively presented goat cheese

* Participants saw Dutch names and a picture without information between parentheses.

† The numbers indicate the fat content of the cheese, based on the percentage of milk fat solids – a common way of indicating cheese differences in the Netherlands.

After making their initial food choices participants were again asked to choose between the nine pairs of products presented earlier, this time on the basis of taste. This time they did not have the option of viewing nutritional information. The aim was to get participants to choose again, solely on the basis of their perception of the anticipated taste. We coded taste preferences as ‘1’ when the unhealthy option was selected, ‘2’ when no preference was indicated and ‘3’ when a preference for the healthy option was indicated. The recorded values per choice were aggregated into a continuous variable ranging from a general taste preference for the unhealthy option (9), to a general taste preference for the healthy option (27).

### Ethical approval

The Open University's ethical review committee reviewed the research proposal. The scope of the research, the informed consent procedure, the contents of the questionnaire and the possible physical and psychological impact on participants were assessed and approved (reference: U2016/03880/FRO).

### Statistical analyses

All analyses were conducted using SPSS Statistics software version 22 (IBM). Participants who did not complete all procedures were excluded from the analyses.

#### Cluster formation

In order to generate groups of consumers based on their nutritional information usage, a cluster analysis was conducted. The variables used for classification included the total frequency and total time of energy, salt, sugar and saturated fat considered. We decided to focus on these four variables, as foods high in these nutrients are generally considered to be unhealthy and thus a risk factor for public health. These variables were standardised into *z*-scores. The analysis was conducted in two steps, using a combination of hierarchical and non-hierarchical clustering approaches, as commonly used in studying food choices^(^^44^^)^. This approach allows us to form clusters with high internal and external homogeneities^(^^45^^)^.

Since hierarchical cluster analyses are sensitive to outliers, univariate outliers on the overall intention to eat healthily score (>1·5 interquartile range; *n* 3) and multivariate outliers on the combined time and frequency of the four nutrition information summary scores (Mahalanobis distance >26·23, *P* < 0·001, *n* 7) were removed from the dataset. This resulted in a total sample of 230 participants for the hierarchical cluster analysis. This cluster analysis was conducted using Ward's method based on squared Euclidian distances. As such, in every step of the clustering process, two clusters are merged such that the squared Euclidean distance between each respondent and the centre of the cluster to which s/he belongs is minimised. The extracted initial cluster centres were saved and used as non-random starting points in an iterative *k*-means clustering procedure^(^^46^^)^. The agglomeration schedule was calculated, and the inverse scree plots of Ward total within-group sums of squared errors of successive cluster solutions were constructed to determine the optimum numbers of clusters (=3).

To examine the stability of the cluster solutions, we used a double-split cross-validation procedure^(^^47^^,^^48^^)^. Following this procedure, the sample was randomly split into halves (subsamples A and B) and the two-step cluster procedure was applied to each half. After that, the participants of subsample A were assigned to new clusters using an iterative *k*-means cluster procedure based on the cluster centres of subsample B and vice versa. The new cluster solutions were then compared for agreement using Cohen's *κ*, to check if the two different approaches resulted in similar clustering solutions. There was very high similarity (*κ* > 0·972). The cluster centres from subsample A had a slightly higher similarity; hence they were used create the definitive cluster solution in the combined dataset.

#### Differences between clusters

Separate one-way ANOVA were conducted for each variable to determine whether there were differences between clusters. Significant differences were subjected to *post hoc* analysis using the Bonferroni or Games–Howell test, depending on the results of a test of homogeneity of variances. A Spearman's rank-order correlation analysis was used to assess cluster differences in percentage of healthy food choices.

#### Multinomial logistic regression

Before carrying out the multinomial logistic regression, we ruled out multicollinearity problems by calculating the variance inflation factor (=1·926). The assumption of proportional odds was met, as assessed by a full likelihood ratio test comparing the fit of the proportional odds model with a model with varying location parameters (*χ*^2^(136) = 73·839; *P* = 1·000). The statistical power of the multinomial logistic regression was assessed based on a binary logistic regression model, because multinomial logistic regression is, in essence, a series of binary logistic regressions. The combined membership of the two smallest clusters (1 and 3; *n* 136) was well above the recommended minimum for empirical validity, namely ten participants per independent variable (seventy, in our study)^(^^49^^)^.

The deviance goodness-of-fit test indicated that the model was a good fit to the observed data (*χ*^2^(428) = 437·825; *P* = 0·361), as did the Pearson goodness-of-fit test (*χ*^2^(428) = 447·107; *P* = 0·253). These results should be treated with some caution, however, as there were a very large number of covariate patterns (230) and 66·7 % of cells had a frequency of zero. A better method of assessing model fit is to compare the fit of the full model with that of an intercept-only model. The difference between these two models with respect to the −2 log likelihood has a *χ*^2^ distribution with df equal to the difference in the number of parameters.

## Results

Three clusters were formed based on patterns in use of information about energy, salt, sugar and saturated fat. Table 2 presents the clusters and the mean values of the variables that were used for cluster formation. We have named the clusters according to the level of use of nutritional information (high, medium, and low).

**Table 2.** Differences between clusters on usage of energy, salt, sugar and saturated fat information considered (both total time and frequency) (Mean values and standard deviations)

	Cluster 1: high information users (*n* 82)		Cluster 2: medium information users (*n* 86)		Cluster 3: low information users (*n* 540)		Total (*n* 222)		
	Mean	sd	Mean	sd	Mean	sd	Mean	sd	*F* (df = 2)
Total frequency energy	30·59^a^	5·60	23·13^b^	5·56	8·93^c^	5·22	22·43	9·97	254·28***
Total time energy (s)	27·14^a^	6·17	17·51^b^	5·24	6·20^c^	3·82	18·32	9·64	254·42***
Total frequency salt	30·01^a^	5·63	20·34^b^	4·89	8·70^c^	5·54	21·08	9·77	261·14***
Total time salt (s)	25·27^a^	6·11	13·86^b^	4·15	5·77^c^	4·39	16·11	9·17	260·41***
Total frequency sugar	28·15^a^	4·91	21·24^b^	4·50	7·54^c^	4·25	20·46	9·16	329·42***
Total time sugar (s)	23·46^a^	6·39	14·23^b^	4·22	5·09^c^	3·57	15·42	8·69	222·69***
Total frequency saturated fat	30·39^a^	5·25	22·23^b^	5·34	8·26^c^	4·38	21·85	9·90	307·98***
Total time saturated fat (s)	28·23^a^	7·15	17·89^b^	4·99	6·07^c^	4·27	18·83	10·27	244·14***

^a,b,c^ Mean values within a row with unlike superscript letters were significantly different (*P* < 0·05; Bonferroni or Games–Howell *post hoc* test).

*** *P* < 0·001.

### Differences between clusters

In line with our overall research question, we compared demographic, psychosocial and other relevant variables in the three clusters. As can be seen in Table 3, the low information users made less healthy choices than those from the other two clusters. A Spearman's test revealed a strong positive correlation between cluster allocation (high *v.* low) and healthy choices (*r*_s_(220) = 0·451; *P* < 0·005). The high information users were older than the other clusters.

**Table 3.** Demographic information, other nutrition information usage and psychosocial variables per cluster (Mean values and standard deviations; percentages)

	Cluster 1: high information users		Cluster 2: medium information users		Cluster 3: low information users		Total		
	Mean	sd	Mean	sd	Mean	sd	Mean	sd	*F*/*χ*^2^ (df = 2)
Food choice behaviour									
Healthy choices (%)	88·95^a^	12·27	84·2^a^	15·39	67·17^b^	18·32	81·81	17·33	35·48***
Demographics									
Age (years)	54·96^a^	12·68	48·93^b^	13·81	49·72^b^	13·62	51·35	13·58	4·81**
BMI (kg/m^2^)	26·10	4·50	25·32	3·79	24·85	3·86	25·49	4·09	1·67
Female (%)	56·1		67·4		63·0		62·2		2·32
Nutrition information									
Total frequency carbohydrates	28·91^a^	5·22	21·13^b^	5·50	8·54^c^	5·92	20·94	9·55	222·99***
Total time carbohydrates (s)	27·23^a^	9·52	15·74^b^	5·95	6·16^c^	5·15	17·65	11·00	139·49***
Total frequency protein	27·93^a^	5·60	21·28^b^	5·96	7·89^c^	4·58	20·48	9·47	216·22***
Total time protein (s)	24·85^a^	6·82	16·77^b^	9·24	5·14^c^	3·71	16·93	10·51	117·90***
Total frequency ingredients	32·24^a^	8·08	24·70^b^	7·37	10·52^c^	7·11	24·04	11·26	134·23***
Total time ingredients (s)	49·48^a^	28·26	29·47^b^	15·20	12·82^c^	12·38	32·81	24·97	53·70***
Total frequency total fat	28·68^a^	5·27	21·08^b^	6·00	8·26^c^	5·40	20·77	9·62	216·98***
Total time total fat (s)	27·02^a^	7·44	15·85^b^	5·60	6·76^c^	6·48	17·76	10·26	162·04***
Psychosocial									
Intention to eat healthily	5·64	0·85	5·66	0·81	5·37	0·88	5·58	0·85	2·33†
Action planning	4·05	1·55	4·03	1·49	3·51	1·64	3·91	1·56	2·45†
Coping planning	4·12	1·54	3·92	1·54	3·49	1·72	3·89	1·60	2·54†
Self-efficacy	5·18	1·06	5·19	1·06	5·25	1·07	5·20	1·06	0·80
Other									
Score on NLS	18·93	2·04	18·76	2·19	18·04	2·89	18·64	2·34	2·55†
Taste preference	20·49^a^	2·35	19·57^b^	2·56	19·09^b^	2·53	19·79	2·53	5·74**
Physical activity	4·32	2·09	4·16	2·05	4·25	2·01	4·24	2·05	0·12
Allergic (%)	14·6		12·8		14·8		14·0		0·16
On diet (%)	28·0		31·4		25·9		28·8		5·81
Tertiary education (%)	74·3		77·7		78·4		76·7		11·98
Perceived importance of attributes									
Ingredients	4·11	0·72	4·06	0·80	3·85	1·05	4·03	0·85	1·62
Energy	3·90^a^	0·88	3·65^a,b^	0·98	3·46^b^	1·19	3·70	1·01	3·28*
Carbohydrates	3·71	0·94	3·74	0·80	3·41	1·09	3·65	0·93	2·45†
Total sugar	4·37^a,b^	0·73	4·47^a^	0·68	4·06^b^	0·92	4·33	0·78	4·95**
Total fat	4·04^a^	0·92	3·94^a,b^	0·87	3·59^b^	1·14	3·89	0·97	3·66*
Saturated fat	4·37^a^	0·76	4·22^a^	0·69	3·61^b^	1·12	4·13	0·89	14·02***
Protein	3·70	0·87	3·69	0·87	3·35	1·01	3·61	0·91	2·85†
Salt	4·45^a^	0·71	4·29^a^	0·73	3·87^b^	0·93	4·25	0·81	9·32***

NLS, Nutrition Literacy Scale.

^a,b,c^ Mean values within a row with unlike superscript letters were significantly different (*P* < 0·05; Bonferroni or Games–Howell *post hoc* test).

* *P* < 0·05, ** *P* < 0·01, *** *P* < 0·001.

† *P* < 0·1.

Interestingly, the three clusters also significantly differed on usage of information on carbohydrates, protein, ingredient declaration and total fat content – all other available information they could consult. Apparently, within our studied health-conscious consumers, there was a high variability in the quantity of information considered, but not what information that was considered. In all instances, the medium information users used significantly less information than the high information users and more information than the low information users.

We observed no univariate differences between clusters with respect to intention to eat healthily, action planning, coping planning and self-efficacy measures (all *P* > 0·05). There were marginal cluster differences (*P* < 0·10) in intention to eat healthily, action planning and coping planning; the low information users appeared to score slightly lower on these psychosocial variables than the other two groups.

Partially in line with our third hypothesis, there was a cluster difference in taste preferences, with high information users showing a higher general taste preference for the healthy options. Unfortunately, we could not confirm the existence of a cluster difference in nutritional literacy scores, although once again there was a marginal difference (*P* < 0·10), with low information users appearing to score slightly lower on a nutritional literacy test than the other two groups.

Furthermore, in line with expectations, we observed a cluster difference in perceptions of the importance of nutritional attributes to the healthiness of food. In general, the high information users perceived information about energy content, total sugar content, total fat content, total saturated fat content and salt content to be more important to the making of healthy choices than the low information users.

### Multinomial logistic regression

Although an ANOVA did not reveal cluster differences in the psychosocial variables (all *P* > 0·05), there were some apparent differences at a descriptive level. We wanted to understand how psychosocial variables contributed to cluster allocation and hence their relationship with use of information about the four nutritional ‘evils’, so we performed a multinomial logistics regression with intention to eat healthily, action planning, coping planning and self-efficacy as covariates and information use as the dependent variable. Because there were cluster differences in age, taste preferences and NLS score we included these variables in our model. Table 4 displays the results of the multinomial logistic regression.

**Table 4.** Effect of age, intention to eat healthily, action planning, coping planning, self-efficacy, score on Nutrition Literacy Scale (NLS) and taste preferences on cluster allocation† (Odds ratios and 95 % Wald confidence intervals)

Cluster	Variable	OR	95 % Wald CI	*B*	*P*
High information users	Age	1·030	1·001, 1·060	0·030	0·041*
	Intention to eat healthily	1·889	1·008, 3·540	0·636	0·047*
	Action planning	1·044	0·699, 1·559	0·043	0·833
	Coping planning	1·388	0·928, 2·076	0·328	0·111
	Self-efficacy	0·404	0·233, 0·700	−0·907	0·001**
	Score on NLS	1·139	0·967, 1·343	0·130	0·120
	Taste preference	1·263	1·079, 1·479	0·234	0·004**
Medium information users	Age	0·996	0·971, 1·022	−0·004	0·774
	Intention to eat healthily	1·880	1·030, 3·430	0·631	0·040*
	Action planning	1·196	0·813, 1·760	0·179	0·363
	Coping planning	1·100	0·757, 1·599	0·095	0·618
	Self-efficacy	0·475	0·280, 0·803	−0·745	0·005**
	Score on NLS	1·119	0·955, 1·312	0·113	0·164
	Taste preference	1·079	0·932, 1·250	0·076	0·309

* *P* < 0·05, ** *P* < 0·01.

† All values are using the low information users as the reference cluster.

In this model, both intention to eat healthily and self-efficacy predicted cluster allocation, which was not apparent from the initial ANOVA (Table 3). Once again, age and taste preference predicted cluster allocation when comparing high information users with low information users. Older people and those who prefer healthier products purely on taste grounds made more use of nutritional information.

The fact that both intention to eat healthily and self-efficacy predicted cluster allocation in this regression model is remarkable. Based on the OR of these two variables, a relatively low intention to eat healthily combined with a relatively high self-efficacy predicts allocation to the low information user cluster. This particular cluster of consumers is less committed to eating healthily than the others in our sample (who had all declared an intention to eat healthily), yet simultaneously is more confident of their ability to make healthy choices.

## Discussion

As explained in the introduction, we set out to investigate possible behavioural, demographic and psychosocial differences between clusters of consumers who intend to eat healthily and have been segmented according to the time they spent looking at information about the energy, salt, sugar and saturated fat content and the frequency with which they consulted such information when making food choices. As expected, our sample of consumers who intended to eat healthily proved to be a heterogeneous group, amongst which there were large differences in use of nutritional information and subsequent food choice behaviour. Between clusters, we observed a 21·78 percentage point difference (88·95 % minus 67·17 %) in how often the healthy option was chosen out of two substitutable food products. With an estimated 200 food decisions daily^(^^50^^)^, over forty-three choices could have resulted in the healthy option if only for cluster allocation.

There were cluster differences in age and taste preferences. Whilst it is possible that the older group may have revisited the cells displaying nutritional information more frequently due to worse memory, it should be noted that the difference in mean age was relatively small (about 6 years; see Table 3). The only cluster to make fewer healthy choices was the low information users. The finding that high information users had a stronger taste preference for the healthy products than low information users may reflect a form of reverse causality. There is evidence that knowing the healthiness of a product influences perceptions of its tastiness^(^^51^^)^, which may explain this result.

More interesting from a public-health perspective is our finding that there is a unique combination of intention to eat healthily and self-efficacy that predicts use of nutritional information. Low information users combined a lower intention to eat healthily with a relatively high self-efficacy when compared with the other clusters. In our sample membership of this cluster was associated with more frequent unhealthy food choices. Confirmation of these results in future studies would suggest that health interventions should focus on avoiding overconfidence in making food decisions, to help ensure that even when consumers’ intention to eat healthily is (temporarily) low, they will make an effort to consult nutritional information prior to making a food choice.

Whilst we agree that improving nutritional literacy should be an important target for public health interventions, this study shows that doing so is likely to have only a limited effect on use of nutritional information. As anticipated, our results show that use of nutritional information to make food choices is a complex phenomenon that is related to more than just nutritional literacy. We therefore recommend that public health campaigns target both nutritional literacy and use of nutritional information to maximise improvement in food choices.

In this respect, proper labelling of the nutritional content of food is of utmost importance to guarantee that consumers can utilise their right to know what is in food products and help them to make better dietary choices. Moreover, we recommend that the abundant availability of foods in general and high accessibility to ultra-processed foods in particular should be reduced to ensure healthier eating across populations^(^^26^^,^^52^^)^.

### Potential interaction between intention to eat healthily and self-efficacy

In recent years many studies have adopted the HAPA model to explain and predict risk-reducing behaviours (e.g. vaccination^(^^53^^)^) and health-enhancing behaviours (e.g. physical activity^(^^21^^)^). In view of its apparent fit to food choice behaviour, an increasing number of studies have focused specifically on the HAPA model in the context of healthy eating^(^^22^^,^^54^^–^^56^^)^. We have provided evidence of its applicability to the prediction of use of nutritional information, which may be considered a healthy behaviour. We would also like to underscore the importance of using multivariate models to understand health behaviour.

There were no cluster differences in the psychosocial factors mentioned in the HAPA model (all *P* > 0·05), although there were some marginal effects (*P* < 0·1). Thus we have no hard evidence that any particular psychosocial factor promotes use of nutritional information and hence cluster allocation. A more in-depth analysis, based on multinomial logistical regression, revealed an interaction between two variables, such that the combination of high intention to eat healthily and low self-efficacy with respect to making healthy food choices had a positive effect on information use. We found that, as in the original HAPA model, motivational and volitional constructs need to be combined to predict an individual's health behaviour.

To investigate this interaction between intention and self-efficacy, we added an interaction variable (intention × self-efficacy) to the multinomial logistics regression model (not presented in Table 4), but this proved non-significant (*P* > 0·622). We then replaced intention to eat healthily with a standardised residual variable that captures only the variance not explained by intention to eat healthily plus self-efficacy. We then replaced intention to eat healthily with a standardised residual variable that takes into account merely the variance, which cannot be explained by both intention to eat healthily and self-efficacy. After computing it using a linear regression of the effect of self-efficacy on intention to eat healthily (*β* = 0·651), we found that self-efficacy became a non-significant predictor in the model (*P* = 0·106). Although we were unable to draw new conclusions from these analyses, they do signal that there is some unaccounted variance that could explain the insignificance of the interaction variable. In future studies, we therefore aim to increase the detail of our measure of a participant's confidence in making healthy food choices. This could help to better understand the phenomenon that there might be a limit to the effect of self-efficacy on the healthiness of food choices^(^^57^^,^^58^^)^.

### Methodological limitations

Whilst this study has several strengths, particularly the use of process-tracing software to capture data on use of nutritional information in the making of food choices and the focus on a neglected sub-population of consumers, those who intend to eat healthily, it also has some limitations. First, it should be noted that the design is cross-sectional, so it is not possible to infer causal relationships from the results; nor does the study give information on long-term outcomes of food choices and the process that precedes the making of choices^(^^59^^)^. Second, in demographic terms our sample was not entirely representative of the general Dutch population, as discussed in our earlier work^(^^18^^)^. Our sample was considerably more educated and had a higher proportion of women than the general Dutch population^(^^60^^)^. The questionnaire was sent out to about 1000 of the email addresses making up the panel, which the panel administrator describes as containing a cross-section of the general population. Third, most individuals generally do not make food choices on a computer (although the number of online grocery shoppers is growing); however, a rapidly growing body of evidence suggests that in social psychological research the differences between outcomes obtained from online convenience samples and laboratory-based off-line research are limited^(^^61^^,^^62^^)^. Fourth, whilst our aim was to present participants with nine pairs of food products that differed only with respect to healthiness, the pairs differed – unsurprisingly – with respect to much more than the nutritional profile. The most important of these differences are those in perceptions of the taste and texture of some of the choices (e.g. spices with *v.* spices without salt, refined bread *v.* whole-wheat bread). There may also have been some differences in the perceived safety of some of the pairs (e.g. frozen string beans *v.* canned beans; processed *v.* unprocessed pork loin). Fifth, we did not use a validated nutrition literacy scale to measure the nutrition literacy construct in the participants of this study. Although Spanish^(^^38^^)^ and Greek^(^^63^^)^ versions of the original English NLS^(^^36^^)^ have been published and validated, no Dutch version has been published and validated to the best of our knowledge. In our translation of the original scale, we took into account the cultural appropriateness and semantic understanding of the questions by our target group. Moreover, we omitted items that were irrelevant to the definition of nutrition literacy as used in this study. We balanced off the empirical and psychometric implications of adapting and shortening a validated questionnaire with the risk of reduced data quality by unclear or irrelevant items. In this respect, a validation of the Dutch items used in this study is warranted through methodologies including structured interviews with participants of this Dutch scale and having participants complete questionnaires related to functional health literacy, similar to the validation studies in Spain and Greece. Lastly, we consider the self-selecting nature of our sample a potential limitation; although all our participants indicated an intention to eat healthily and we have confirmed this through our questionnaire, some segments of the population of consumers who intend to eat healthily may have been under-represented in our sample, particularly those who are less expressive of their intention.

Our cluster analysis focused on use of information about the salt, sugar, saturated fat and energy content of food products. As noted in the introduction, there is strong evidence that excessive intake of foods high in these four ‘evils’ is a public health problem. One could argue that we could have clustered on all nutrition information considered. Given the high and significant differences between clusters on all other nutrition information (carbohydrates, protein, ingredient declaration, total fat), however, our results will probably not change significantly. It is also this correlation that barred us from clustering on differences between what information was considered, rather than the overall amount that is considered. It would be interesting to disentangle those consumers who focus only on salt, for instance, from those who focus only on sugar. Our current methodology, however, did not allow such cluster formation.

In this context, it is also interesting to consider those participants who made extremely limited use of nutritional information in our experiment. As the name and picture of the product were always visible, participants may have obtained a considerable amount of information from these sources alone. In a future study, it would be worth investigating how participants infer nutritional information from pictures and product names. The literature on heuristics provides evidence of a ‘less is more’ effect when it comes to nutritional information^(^^39^^,^^41^^,^^64^^)^. The attenuating role of self-efficacy in our study suggests an avenue for further study in this field.

### Recommendations and conclusion

Using data from our online process tracing study we were able to unravel the heterogeneity of consumers who intend to eat healthily and link this to their food choices. We found that within this group there are clusters of consumers who often choose the less healthy option, perhaps without being aware that they are doing so. As hypothesised, when clustered according to the duration and frequency of viewing of information about the energy, salt, sugar and saturated fat content of food products, we found cluster differences in food choices (hypothesis 1).

Our in-depth analysis revealed some evidence that cluster membership is influenced by an interaction between motivational and volitional constructs, particularly intention to eat healthily and self-efficacy, partially confirming hypothesis 2. Although our results are not conclusive, they indicate that it may be worth considering a shift in the focus of public health interventions. The case of such a shift would be stronger if the interaction between intention and self-efficacy (i.e. low intention combined with high self-efficacy that results in less usage of nutritional values) is also observed in consumers who do not express a particular intention to eat healthily. It could very well be that interventions designed to promote healthy eating result in some consumers becoming overconfident about their ability to make healthy choices and hence lead to less healthy choices. Public health messages might need to be tailored to specific subgroups to ensure that – regardless of good intentions and confidence – people still consult nutritional information prior to making food choices. For this, we recommend replicating our study in a different population. In such a study, an improved measure for self-efficacy should be used to understand better the unaccounted variance in our model.

In our previous study^(^^18^^)^ we argued that models based on motivational and volitional constructs should be augmented to improve their predictive power. We have found, for instance, that nutritional literacy is an important predictor of the percentage of healthy choices made. In this study we used cluster analysis to demonstrate that taste preferences played an important role in food choices (hypothesis 3), whereas nutritional literacy had a limited effect on use of nutritional information and hence cluster allocation.

Our study shows that consumers who intend to eat healthily cannot be treated as a single population, and this should be reflected in the approaches taken by public health interventionists who are targeting this important group. A more tailored approach to communication and guidance is required. There are huge differences within this group in terms of use of nutritional information and subsequent food choices. We recommend that these differences should be taken into account in the development of innovative, evidence-based policies and interventions to promote healthier food choices.

## References

[1] O'Neill S & O'Driscoll L (2015) Metabolic syndrome: a closer look at the growing epidemic and its associated pathologies. Obes Rev 16, 1–12. 10.1111/obr.12229 25407540

[2] Monteiro CA, Cannon G, Moubarac J-C, (2018) The UN Decade of Nutrition, the NOVA food classification and the trouble with ultra-processing. Public Health Nutr 21, 5–17. 28322183 10.1017/S1368980017000234 PMC10261019

[3] Hoefkens C, Verbeke W & Van Camp J (2011) European consumers’ perceived importance of qualifying and disqualifying nutrients in food choices. Food Qual Prefer 22, 550–558.

[4] Van Buul VJ & Brouns FJPH (2015) Nutrition and health claims as marketing tools. Crit Rev Food Sci Nutr 55, 1552–1560. 24364816 10.1080/10408398.2012.754738

[5] He FJ, Brinsden HC & MacGregor GA (2014) Salt reduction in the United Kingdom: a successful experiment in public health. J Hum Hypertens 28, 345–352. 24172290 10.1038/jhh.2013.105

[6] Ellison B, Lusk JL & Davis D (2012) Effect of menu labeling on caloric intake and restaurant revenue in full-service restaurants. Selected paper for presentation at the AAEA Annual Meeting, Seattle.

[7] Grunert KG, Fernández-Celemín L, Wills JM, (2010) Use and understanding of nutrition information on food labels in six European countries. J Public Health (Bangkok) 18, 261–277. 10.1007/s10389-009-0307-0 PMC2967247 21124644

[8] Kozup JC, Creyer EH & Burton S (2003) Making healthful food choices: the influence of health claims and nutrition information on consumers’ evaluations of packaged food products and restaurant menu items. J Mark **67**, 19–34.

[9] Ebneter DS, Latner JD & Nigg CR (2013) Is less always more? The effects of low-fat labeling and caloric information on food intake, calorie estimates, taste preference, and health attributions. Appetite 68, 92–97. 23632034 10.1016/j.appet.2013.04.023

[10] Mhurchu CN, Volkova E, Jiang Y, (2017) Effects of interpretive nutrition labels on consumer food purchases: the Starlight randomized controlled trial. Am J Clin Nutr 105, 695–704. 28148503 10.3945/ajcn.116.144956

[11] Bornkessel S, Bröring S, Omta SWF, (2014) What determines ingredient awareness of consumers? A study on ten functional food ingredients. Food Qual Prefer 32, 330–339.

[12] Ellison B, Lusk JL & Davis D (2013) Looking at the label and beyond: the effects of calorie labels, health consciousness, and demographics on caloric intake in restaurants. Int J Behav Nutr Phys Activ 10, 21. 10.1186/1479-5868-10-21 PMC3598881 23394433

[13] Mai R & Hoffmann S (2015) How to combat the unhealthy=tasty intuition: the influencing role of health consciousness. J Public Policy Mark 34, 63–83.

[14] Wardle J (1993) Food choices and health evaluation. Psychol Health 8, 65–75.

[15] Brannon L, Feist J & Updegraff J (2014) Health Psychology: An Introduction to Behavior and Health, 8th ed. Belmont, CA: Wadsworth Cengage Learning.

[16] Sutton S (2005) Stage theories of health behaviour In Predicting Health Behaviour: Research and Practice with Social Cognition Models, 2nd ed., pp. 223–275 [M Conner and P Norman, editors]. Buckingham: Open University Press.

[17] Rothman AJ, Gollwitzer PM, Grant AM, (2015) Hale and hearty policies: how psychological science can create and maintain healthy habits. Perspect Psychol Sci 10, 701–705. 26581721 10.1177/1745691615598515

[18] Van Buul VJ, Bolman CAW, Brouns FJPH, (2017) Back-of-pack information in substitutive food choices: a process-tracking study in participants intending to eat healthy. Appetite 116, 173–183. 28472643 10.1016/j.appet.2017.04.036

[19] Wahlich C, Gardner B & McGowan L (2013) How, when and why do young women use nutrition information on food labels? A qualitative analysis. Psychol Health 28, 202–216. 22924452 10.1080/08870446.2012.716439

[20] Schwarzer R (editor) (1992) Self-efficacy in the adoption and maintenance of health behaviours In Theoretical Approaches and a New Model, pp. 217–243. Washington, DC: Hemisphere Publishing Corp.

[21] Lippke S & Plotnikoff RC (2014) Testing two principles of the health action process approach in individuals with type 2 diabetes. Health Psychol 33, 77. 23088172 10.1037/a0030182

[22] Godinho CA, Alvarez M & Lima ML (2013) Formative research on HAPA model determinants for fruit and vegetable intake: target beliefs for audiences at different stages of change. Health Educ Res 28, 1014–1028. 23856178 10.1093/her/cyt076

[23] Springvloet L, Lechner L & Oenema A (2014) Can individual cognitions, self-regulation and environmental variables explain educational differences in vegetable consumption?: a cross-sectional study among Dutch adults. Int J Behav Nutr Phys Act 11, 149. 25480542 10.1186/s12966-014-0149-1 PMC4275939

[24] Carbone ET & Zoellner JM (2012) Nutrition and health literacy: a systematic review to inform nutrition research and practice. J Acad Nutr Diet 112, 254–265. 22732460 10.1016/j.jada.2011.08.042

[25] Rothman RL, Housam R, Weiss H, (2006) Patient understanding of food labels: the role of literacy and numeracy. Am J Prev Med 31, 391–398. 17046410 10.1016/j.amepre.2006.07.025

[26] Pinho MGM, Mackenbach JD, Oppert JM, (2018) Exploring absolute and relative measures of exposure to food environments in relation to dietary patterns among European adults. Public Health Nutr (epublication ahead of print version 7 December 2018). 10.1017/S1368980018003063 PMC6536821 30523774

[27] Raghunathan R, Naylor RW & Hoyer WD (2006) The unhealthy=tasty intuition and its effects on taste inferences, enjoyment, and choice of food products. J Mark 70, 170–184.

[28] Pendergast D, Garvis S & Kanasa H (2011) Insight from the public on home economics and formal food literacy. Fam Consum Sci Res J 39, 415–430.

[29] Velardo S (2015) The nuances of health literacy, nutrition literacy, and food literacy. J Nutr Educ Behav 47, 385–389.e1. 26026651 10.1016/j.jneb.2015.04.328

[30] Yuen EYN, Thomson M & Gardiner H (2018) Measuring nutrition and food literacy in adults: a systematic review and appraisal of existing measurement tools. Health Literacy Res Pract 2, e134–e160. 10.3928/24748307-20180625-01 PMC6607839 31294289

[31] Schwarzer R & Renner B (2000) Social–cognitive predictors of health behavior: action self-efficacy and coping self-efficacy. Health Psychol 19, 487. 11007157

[32] Schwarzer R, Schüz B, Ziegelmann JP, (2007) Adoption and maintenance of four health behaviors: theory-guided longitudinal studies on dental flossing, seat belt use, dietary behavior, and physical activity. Ann Behav Med 33, 156–166. 17447868 10.1007/BF02879897

[33] Sniehotta FF, Scholz U & Schwarzer R (2006) Action plans and coping plans for physical exercise: a longitudinal intervention study in cardiac rehabilitation. Br J Health Psychol 11, 23–37. 16480553 10.1348/135910705X43804

[34] Luszczynska A, Tryburcy M & Schwarzer R (2007) Improving fruit and vegetable consumption: a self-efficacy intervention compared with a combined self-efficacy and planning intervention. Health Educ Res 22, 630–638. 17060349 10.1093/her/cyl133

[35] Nguyen TH, Park H, Han H, (2015) State of the science of health literacy measures: validity implications for minority populations. Patient Educ Couns 98, 1492–1512. 10.1016/j.pec.2015.07.013 PMC4732928 26275841

[36] Diamond JJ (2007) Development of a reliable and construct valid measure of nutritional literacy in adults. Nutr J 6, 1475–2891. 10.1186/1475-2891-6-5 PMC1804274 17300716

[37] Haun JN, Valerio MA, McCormack LA, (2014) Health literacy measurement: an inventory and descriptive summary of 51 instruments. J Health Commun 19, 302–333. 25315600 10.1080/10810730.2014.936571

[38] Coffman MJ & La-Rocque S (2012) Development and testing of the Spanish Nutrition Literacy Scale. Hisp Health Care Int 10, 168–174.

[39] Scheibehenne B, Miesler L & Todd PM (2007) Fast and frugal food choices: uncovering individual decision heuristics. Appetite 49, 578–589. 17531348 10.1016/j.appet.2007.03.224

[40] Willemsen MC & Johnson EJ (2011) Visiting the decision factory: observing cognition with MouselabWEB and other information acquisition methods In A Handbook of Process Tracing Methods for Decision Research: A Critical Review and User's Guide, pp. 21–42 [M Schulte-Mecklenbeck, A Kühberger and R Ranyard, editors]. New York: Psychology Press.

[41] Schulte-Mecklenbeck M, Sohn M, de Bellis E, (2013) A lack of appetite for information and computation. Simple heuristics in food choice. Appetite 71, 242–251. 23994507 10.1016/j.appet.2013.08.008

[42] Just MA & Carpenter PA (1980) A theory of reading: from eye fixations to comprehension. Psychol Rev 87, 329. 7413885

[43] Voedingscentrum (2016) Wat staat niet in de Schijf van vijf? (What is not in the Disc of five?) http://www.voedingscentrum.nl/nl/gezond-eten-met-de-schijf-van-vijf/hoeveel-en-wat-kan-ik-per-dag-eten-/wat-staat-niet-in-de-schijf-van-vijf-.aspx (accessed July 2016).

[44] Gorton M, Ness M & White J (2013) Segmenting consumers using cluster analysis: an application to food motivations in the Western Balkan countries In Food Consumer Science: Theories, Methods and Application to the Western Balkans, pp. 43–55 [D Barjolle, M Gorton, J Milošević Đorđević and Ž Stojanović, editors]. Dordrecht: Springer Netherlands.

[45] Hair JF & Black WC (2000) Cluster analysis In Reading and Understanding More Multivariate Statistics, pp. 99–146 [LG Grimm and PR Yarnold, editors]. Washington, DC: American Psychological Association.

[46] Clatworthy J, Hankins M, Buick D, (2007) Cluster analysis in illness perception research: a Monte Carlo study to identify the most appropriate method. Psychol Health 22, 123–142.

[47] Friederichs SAH, Bolman CAW, Oenema A, (2015) Profiling physical activity motivation based on self-determination theory: a cluster analysis approach. BMC Psychol 3, 1. 25678981 10.1186/s40359-015-0059-2 PMC4310201

[48] Vansteenkiste M, Sierens E, Soenens B, (2009) Motivational profiles from a self-determination perspective: the quality of motivation matters. J Educ Psychol 101, 671.

[49] Hosmer DW Jr, Lemeshow S & Sturdivant RX (2013) Applied Logistic Regression, 3rd ed. Hoboken, NJ: John Wiley & Sons, Inc.

[50] Wansink B & Sobal J (2007) Mindless eating: the 200 daily food decisions we overlook. Environ Behav 39, 106–123.

[51] Werle COC, Trendel O & Ardito G (2013) Unhealthy food is not tastier for everybody: The “healthy=tasty” French intuition. Food Qual Prefer 28, 116–121.

[52] Swinburn BA, Sacks G, Hall KD, (2011) The global obesity pandemic: shaped by global drivers and local environments. Lancet 378, 804–814. 21872749 10.1016/S0140-6736(11)60813-1

[53] Ernsting A, Knoll N, Schneider M, (2015) The enabling effect of social support on vaccination uptake via self-efficacy and planning. Psychol Health Med 20, 239–246. 24862090 10.1080/13548506.2014.920957

[54] Richert J, Reuter T, Wiedemann AU, (2010) Differential effects of planning and self-efficacy on fruit and vegetable consumption. Appetite 54, 611–614. 20227450 10.1016/j.appet.2010.03.006

[55] Lange D, Richert J, Koring M, (2013) Self-regulation prompts can increase fruit consumption: a one-hour randomised controlled online trial. Psychol Health 28, 533–545. 23282217 10.1080/08870446.2012.751107

[56] Radtke T, Kaklamanou D, Scholz U, (2014) Are diet-specific compensatory health beliefs predictive of dieting intentions and behaviour? Appetite 76, 36–43. 24472827 10.1016/j.appet.2014.01.014

[57] Huntsinger ET & Luecken LJ (2004) Attachment relationships and health behavior: the mediational role of self-esteem. Psychol Health 19, 515–526.

[58] Hartmann C, Dohle S & Siegrist M (2015) A self-determination theory approach to adults’ healthy body weight motivation: a longitudinal study focussing on food choices and recreational physical activity. Psychol Health 30, 924–948. 25584714 10.1080/08870446.2015.1006223

[59] Berkman ET (2018) Value-based choice: an integrative, neuroscience-informed model of health goals. Psychol Health 33, 40–57. 28403660 10.1080/08870446.2017.1316847 PMC5897039

[60] Centraal Bureau voor de Statistiek (2016) Statline. http://statline.cbs.nl/Statweb/ (accessed April 2019).

[61] Riva G, Teruzzi T & Anolli L (2003) The use of the Internet in psychological research: comparison of online and offline questionnaires. Cyberpsychol Behav 6, 73–80. 12650565 10.1089/109493103321167983

[62] Casler K, Bickel L & Hackett E (2013) Separate but equal? A comparison of participants and data gathered via Amazon's MTurk, social media, and face-to-face behavioral testing. Comput Human Behav 29, 2156–2160.

[63] Michou M, Panagiotakos DB & Costarelli V (2019) Development & validation of the Greek version of the nutrition literacy scale. Med J Nutrition Metab 12, 61–67.

[64] Wansink B, Just D & Payne CR (2009) Mindless eating and healthy heuristics for the irrational. Am Econ Rev 99, 165. 29505211 10.1257/aer.99.2.165

